# A giant ovarian mass

**DOI:** 10.11604/pamj.2024.47.172.43287

**Published:** 2024-04-09

**Authors:** Haithem Aloui, Rachid Hentati

**Affiliations:** 1 Department of Gynecology and Obstetrics of Tunis Maternity and Neonatology Center, University of Tunis El Manar, Faculty of Medicine of Tunis, Tunis, Tunisia

**Keywords:** Giant mass, serous cystadenoma, ovary

## Image in medicine

It was a 36-year-old patient with no significant medical history. She presented with rapidly progressive abdominal distension and chronic pelvic pain without other associated symptoms. On examination, the abdomen was soft but distended, with filling of the left vaginal cul-de-sac without pain upon uterine mobilization. Ultrasound revealed a mass occupying the entire abdominal cavity, with finely echogenic, multiseptated fluid content, no endocystic vegetation, and non-vascularized on Doppler. Pelvic magnetic resonance imaging (MRI) showed a 35cm mass with multiple septations, multiple papillary cystic projections at the margins, non-enhancing after left ovarian injection classified as ovarian-adnexal reporting and data system (ORADS-4). Tumor markers: Alpha-fetoprotein, CA19-9, and ACE were requested and returned negative, while CA125 marker was positive at 52 U/mL. During surgery, the mass appeared whitish, with a dual solid-cystic component, vascularized, adherent to the posterior peritoneum. Homolateral annexectomy was performed along with cytology of peritoneal fluid and peritoneal biopsies. The pathological examination of the operative specimen revealed a 40cm structure filled with greenish fluid, having thickened and richly vascularized lining with areas of fibrosis. It was lined by a single-layered epithelium consisting of small, regular nuclei with scant cytoplasm. The diagnosis concluded with a serous cystadenoma, without signs of malignancy. The postoperative course was uneventful, and the patient was discharged on postoperative day 3.

**Figure 1:**
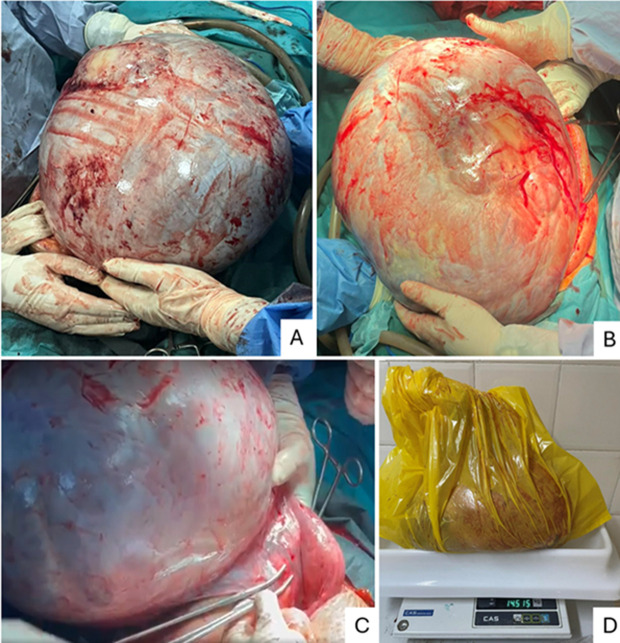
A, B, C, D) giant ovarian mass

